# Lasting mesothalamic dopamine imbalance and altered exploratory behavior in rats after a mild neonatal hypoxic event

**DOI:** 10.3389/fnint.2023.1304338

**Published:** 2024-01-17

**Authors:** Barbara Nikolic, Sara Trnski-Levak, Kristina Kosic, Matea Drlje, Ivan Banovac, Dubravka Hranilovic, Natasa Jovanov-Milosevic

**Affiliations:** ^1^Department of Biology, University of Zagreb Faculty of Science, Zagreb, Croatia; ^2^Croatian Institute for Brain Research, University of Zagreb School of Medicine, Zagreb, Croatia; ^3^Department of Biology, University of Zagreb School of Medicine, Zagreb, Croatia; ^4^Department for Anatomy and Clinical Anatomy, University of Zagreb School of Medicine, Zagreb, Croatia

**Keywords:** catecholamine, brainstem, thalamus, non-invasive model, DARPP-32, cognitive impairment

## Abstract

**Introduction:**

Adversities during the perinatal period can decrease oxygen supply to the fetal brain, leading to various hypoxic brain injuries, which can compromise the regularity of brain development in different aspects. To examine the catecholaminergic contribution to the link between an early-life hypoxic insult and adolescent behavioral aberrations, we used a previously established rat model of perinatal hypoxia but altered the hypobaric to normobaric conditions.

**Methods:**

Exploratory and social behavior and learning abilities were tested in 70 rats of both sexes at adolescent age. Inherent vertical locomotion, sensory-motor functions and spatial learning abilities were explored in a subset of animals to clarify the background of altered exploratory behavior. Finally, the concentrations of dopamine (DA) and noradrenaline in midbrain and pons, and the relative expression of genes for DA receptors D1 and D2, and their down-stream targets (DA- and cAMP-regulated phosphoprotein, Mr 32 kDa, the regulatory subunit of protein kinase A, and inhibitor-5 of protein phosphatase 1) in the hippocampus and thalamus were investigated in 31 rats.

**Results:**

A lesser extent of alterations in exploratory and cognitive aspects of behavior in the present study suggests that normobaric conditions mitigate the hypoxic injury compared to the one obtained under hypobaric conditions. Increased exploratory rearing was the most prominent consequence, with impaired spatial learning in the background. In affected rats, increased midbrain/pons DA content, as well as mRNA levels for DA receptors and their down-stream elements in the thalamus, but not the hippocampus, were found.

**Conclusion:**

We can conclude that a mild hypoxic event induced long-lasting disbalances in mesothalamic DA signaling, contributing to the observed behavioral alterations. The thalamus was thereby indicated as another structure, besides the well-established striatum, involved in mediating hypoxic effects on behavior through DA signaling.

## 1 Introduction

There has been growing evidence that multiple epigenetic factors during gestation and the perinatal period modulate brain development, shaping individual susceptibility to various neuropsychiatric disorders later in life (Faa et al., 2014). Changes in the maternal environment or placental dysfunction may lead to lowered fetal oxygen supply, often combined with reduced blood supply, and seriously affect the developing neuronal tissue, leading to a hypoxic brain injury (HBI; Frajewicki et al., 2020; Piešová and Mach, 2020). Severe HBI, induced by perinatal hypoxia-ischemia, could result in the infant’s death or permanent neurologic deficits, such as motor disabilities, seizures, impaired muscle tone, and epilepsy (Leviton and Nelson, 1992; Pin et al., 2009). On the other hand, moderate or mild HBI may pass unnoticed at birth but could still affect normal brain development and manifest later in childhood or adolescence as cognitive problems or behavioral disorders (Gonzalez and Miller, 2006; Nalivaeva et al., 2018).

To unravel events between an early life insult and development of behavioral symptoms later in life, and to discover the underlying mechanisms of HBI, various animal models have been employed. The hypoxia-ischemia (HI) models, which combine surgically induced ischemia with exposure to a hypoxic condition, anatomically and functionally correspond to the more severe HBI in humans and are mainly used for modeling neurological disorders. The “hypoxia-only” (H) models employ acute or chronic exposure to hypoxic conditions that do not induce necrosis, correspond to a mild hypoxic injury in human fetuses and prematurely born babies, and are suitable for exploring biochemical, molecular, and structural events, leading to psychiatric/behavioral disorders (Millar et al., 2017; Hefter et al., 2018).

The exploratory behavior of a rodent in a novel environment is based on an evolutionary balance between a need to secure resources and a need to minimize the risk of predation (Thompson et al., 2018). Rearing is an essential aspect of exploratory behavior that combines sensory perception, spatial memory acquisition, and vigilance. There are two types of rearing—supported (wall-leaning) and unsupported (brief rise on hind legs), both considered to be valuable tools for collecting information about the novel space (Lever et al., 2006). Unsupported rearing provides an animal with visual information needed for spatial mapping, while supported rearing, through somatosensory perception, enables the acquisition of information about environmental boundaries (Crusio, 2001; Lever et al., 2006; Sturman et al., 2018).

The thalamus seems to bridge sensory perception and cognition with an aim to identify environmental signals bearing current behavioral relevance (Wolff et al., 2021). Some thalamic nuclei convey sensory information from the site of reception to the area of perception, playing an important role in their filtering and organization (Sherman, 2005). Other thalamic nuclei send projections to the cingulate cortex and hippocampal formation (Shibata, 1993; van Groen and Wyss, 1995; Aggleton et al., 2010) and seem to be involved in spatial learning and memory (Aggleton et al., 1991; Savage et al., 1998; van Groen et al., 2002). The thalamic relay function can be modulated depending on behavioral demands, and important modulators are the brainstem monoaminergic neurotransmitters, including dopamine (DA; Venkatraman et al., 2017).

The DA system is highly conserved among vertebrates, bearing a crucial role in the adaptation of animal behavior (Yamamoto and Vernier, 2011). The primary source of DA in the brain are the midbrain DA neurons, which send projections to various parts of the forebrain through the three main dopaminergic pathways—nigrostriatal, mesolimbic, and mesoprefrontal (Islam et al., 2021). In primates, dopaminergic afferents also project from the ventral tegmental area (VTA), periaqueductal gray, and the lateral parabrachial nucleus broadly across the thalamus (Sánchez-González et al., 2005). To a lesser extent, dopaminergic innervation of the thalamus (Papadopoulos and Parnavelas, 1990; García-Cabezas et al., 2009; Varela, 2014) as well as the presence of dopamine receptor 1 (D1; Fremeau et al., 1991; Huang et al., 1992) and dopamine receptor 2 (D2) (Khan et al., 1998; Clark et al., 2017) have also been demonstrated in rats. The binding of DA to the D1 receptor activates (via stimulatory G-protein) the effector adenylate-cyclase, raising cAMP levels and activating protein kinase A (PKA). PKA then phosphorylates two proteins: dopamine- and cAMP-regulated phosphoprotein Mr. 32 kDa (DARPP-32) and inhibitor-5 of protein phosphatase 1 (IPP5), thereby turning them into potent inhibitors of serine/threonine protein phosphatase (PP-1), which plays a significant role in dephosphorylation of eukaryotic cell proteins (Walaas et al., 2011). The binding of DA to the D2 receptor elicits the opposite effect, inhibiting the PKA/DARPP-32/PP-1 signaling.

Expression of DA receptors occurs early in development and plays an important role in shaping neuronal cytoarchitecture. During this period, various disruptors can alter DA signaling, affecting brain structure and connectivity (Money and Stanwood, 2013). Post-mortem analysis of human neonates indicated a vulnerability of mesencephalic DA neurons to prolonged neonatal hypoxia (Pagida et al., 2013), while the study on a rodent model showed that perinatal hypoxia affected early differentiation of mesencephalic DA neurons and postnatal brain DA terminal architecture (Brandon et al., 2022). These findings indicate DA dysfunction as one of the consequences of perinatal HBI that can later lead to the development of DA-related behavioral and/or cognitive deficits in infant survivors (Giannopoulou et al., 2018).

In H models, hypoxia is achieved by placing an animal in a chamber with either decreased barometric pressure (hypobaric hypoxia, HH) or reduced oxygen fraction (normobaric hypoxia, NH). Although both procedures decrease partial oxygen pressure, physiological studies on adult animals and humans revealed a more intense reaction to HH than to NH, concerning generalized hypoxemia, hypocapnia, blood alkalosis, or arterial oxygen saturation (Savourey et al., 2003). In an attempt to compare the consequences of perinatal exposure to HH and NH and to choose the more suitable animal model for further studies, we have exposed postnatal day 1 (P1) pups to both modes of hypoxia. In our previous study, we explored a HH model and observed a long-lasting reorganization of the connectivity in the cingulate cortex, accompanied by cognitive impairment and prominent alteration in exploratory behavior that persisted in adulthood (Trnski et al., 2022).

In the present study, we exposed P1 neonates to a mild NH. We hypothesized the following: (1) Milder behavioral alterations after the exposure to NH, compared to HH, could be expected, but some aspects of altered exploratory behavior, as the most prominent behavioral consequence of hypobaric HBI, should be preserved, (2) the altered exploratory behavior could be related to the impairments in sensory processing and spatial learning, and (3) the increased DA and/or noradrenaline (NA) concentrations in the midbrain/pons could be expected, affecting thereby downstream signaling in the regions involved in somatosensory processing and spatial learning (i.e., thalamus and hippocampus). To compare behavioral consequences of NH to that of HH, we submitted a large group of hypoxic and control animals of adolescent age to a set of behavioral tests investigating exploratory behavior, social behavior, and learning. In order to search for the functional background of altered exploratory behavior, we then submitted another group of animals to a set of tests specifically exploring sensory-motor function and spatial learning. Finally, to check neurochemical alterations induced by hypoxia in subcortical brain regions involved in the mediation of exploratory behavior, we measured catecholamine concentrations in the part of the brain stem encompassing the midbrain (the main source of DA) and pons (the main source of NA). Additionally, we assessed the relative expression of genes for dopamine D1 and D2 receptors and their downstream targets in the hippocampus and thalamus. Animals of both sexes were used to check the potential differential vulnerability to HBI.

## 2 Materials and methods

### 2.1 Animals

All animal experiments comply with the ARRIVE guidelines and have been carried out following the United Kingdom Animals (Scientific Procedures) Act, 1986, EU Directive 2010/63/EU, Croatian regulations for experimentation on animals, and constitutive documents: NN 102/2017 and 32/19; NN 55/2013; 39/17 and 116/2019. The ethical committee of the University of Zagreb and national ethical and animal welfare bodies (EP231/2019; UP/I-322-01/19.01/75) approved the study design and experiments. Every effort was made to reduce the number of animals in use and to minimize animal discomfort.

#### 2.1.1 Animal housing

Animals were housed in polysulfone cages within a controlled environment with a temperature of 21 ± 2°C and humidity maintained at 65 ± 5%. A light or dark cycle of 12:12 h was employed with the light period beginning at 7 a.m. Food (4RF21C, Mucedola srl, Settimo Milanese MI, Italy) and tap water were provided *ad libitum*. The minimum number of animals needed for the study was determined by power analysis, and a total of 102 1-day-old Wistar Han (RccHan: WIST) rats were obtained from our breeding facility (School of Medicine, University of Zagreb, Croatia). The day of birth was considered P0 until noon of the next day when P1 began. After exposure to hypoxia, pups were permanently marked by a toe tattoo (NEO-9 Neonate Tattoo System, AgnTho’s AB, Sweden) and returned to dams until weaning at P30. After weaning, they were separated by sex and group, and 3–4 of them were kept in each cage until they were sacrificed for brain tissue collection (P50). A scheme of experiments and number of animals used can be found in Table 1.

**Table 1:** Experimental design.

Experiment	Age	Number of animals		
	Postnatal day (P)	Experimental	Control	Total
Initial behavioral tests	P33–P43	36 (18F, 18 M)	34 (18F, 16 M)	70 (36F, 34 M)
*Open field*	*P33*			
*Hole board*	*P35*			
*T-maze*	*P37–P41*			
*Social choice*	*P43*			
**Subgroup of rats**				
Tissue collection for ELISA and qPCR	P50	16 (8F, 8 M)	15^*^ (8F, 7 M)	31 (16F, 15 M)
**New set of animals**				
Additional behavioral tests	P33–P41	16 (8F, 8 M)	16 (8F, 8 M)	32 (16F, 16 M)
*Cylinder test*	*P33*			
*Object location memory*	*P34–P36*			
*Adhesive tape removal*	*P37–P41*			

^*^Due to the extreme passiveness in all behavioral tests, one rat was eliminated from further study.

#### 2.1.2 Exposure of animals to normobaric hypoxia

P1 pups (without any dysmorphic features and of an average body mass of 6.68 ± 0.27 g) were randomly assigned to the hypoxic or control group, keeping both sexes equally represented. A modification of the hypoxia-inducing protocol was made according to Zhang et al. (2013). The hypoxic group (4F + 4 M per session) was placed for 2 h in a closed-heated hypoxia chamber (STEMCELL Technologies Inc. Vancouver, Canada; Cat. No. 27310) connected to the bottle containing the calibration gas mixture of 8% O_2_ and 92% N_2_ (Messer Croatia Plin d.o.o., Zaprešić, Croatia), using a spectromed cylinder pressure regulator FM41-S1 (Spectron Gas Control Systems GmbH, Langen, Germany), which enabled a continuous flow of a mixture of gases of 3.5 L/min. During the first 15 min, temperature was gradually raised from 23.4 to 30°C and then maintained at 30°C until the end of the experiment. For animal comfort and welfare, webs of cellulose fibers and bedding from the home cage were placed in the chamber. Oxygen partial pressure, temperature, pressure, and humidity were continuously monitored by an optical oxygen gas sensor—FDO2 (PyroScience GmbH, Aachen, Germany) connected to a computer. An absorbent for CO₂ was also placed in the chamber to annulate CO_2_ exhaled by the animals. The control group (4F + 4 M per session) was placed in a smaller cage at normoxic conditions (atmospheric air 21% O_2_ and 79% N_2_) in the above described heating regime for 2 h together with bedding from a home cage and a thermometer/hygrometer.

### 2.2 Behavioral testing

Testing was performed between 2 and 6 pm under illumination of 30 lx, in a randomized order, by experimenters unaware of the rat’s treatment group. The apparatuses were thoroughly cleaned after each animal or each trial to remove odor. Sessions were filmed using a camera placed above the apparatus and analyzed using EthoVisionXT13 software (Noldus Information Technology Inc., Leesburg, VA, SAD).

#### 2.2.1 Initial behavioral tests

A set of initial behavioral tests was performed on 34 control (18 female rats and 16 male rats) and 36 hypoxic (18 female rats and 18 male rats) animals, from P33 to P43, in the following order: open field, hole-board test, T-maze, and social choice. Apparatuses and procedures have been described in detail in our previous research (Blazevic et al., 2012; Trnski et al., 2022).

An open field (of) enclosure was used to measure a novel space exploration behavior consisting of horizontal movement interrupted by occasional stopping, head scanning, and rearing. It is influenced by the inherent locomotor activity reflected as ambulation in an open field, exploratory tendency reflected as rearing frequency, and anxiety level reflected as the amount of time spent on a less safe location of the testing apparatus (Thompson et al., 2018). Distance covered (DCof in cm), time spent in movement (TMof in s), and number of rearings (Rof) were recorded during a 5-min session.

Same enclosure was turned into a hole board (hb) and used to study head dipping (both eyes in a hole) as a form of novel object exploration behavior involving orienting toward, touching, and sniffing a novel object, which provides animal information on the potential usefulness or threat of an unfamiliar item (Brown and Nemes, 2008). The total number of holes visited (THVhb) and the percentage of inner holes (%INhb) were recorded during a 5-min session.

Reward-motivated spatial learning was tested in a T-maze (tm) as an attempt to find a reward (food pellet) according to a Win/Stay strategy. The number of correct choices (CCtm) in sessions of 10 60-s trials during 5 consecutive days was recorded. During the entire testing period, rats had access to food only for 1 h daily after the session.

Sociability represents a degree of interest in an unknown conspecific and can be measured as the time spent exploring a conspecific in a social choice test (sc). A testing rat was placed into the central compartment and, after 3 min of habituation, was allowed to freely explore side compartments, containing either an inanimate object or a same-sex conspecific placed in a wire, during a 5-min period. Latency to approach the rat (LRsc) and time spent exploring the rat (TRsc) were recorded as measures of sociability. Time out of the middle chamber (TOMCsc in s), number of rearings (Rsc), and time spent exploring an object (TOsc) were recorded as additional measures of anxiety level, novel space exploration, and novel object exploration, respectively.

#### 2.2.2 Additional behavioral tests

To check which functional aspect of exploratory rearing has been potentially changed, additional tests were performed on another group of 16 control (eight female rats and eight male rats) and 16 hypoxic (eight female rats and eight male rats) animals, from P33 to P41, in the following order: cylinder test, object location memory, and adhesive removal test.

The level of spontaneous vertical locomotion was examined in the cylinder test (CT), developed for assessing forelimb use asymmetry during spontaneous rearing in rodent models of neurological deficits (Schallert et al., 2000; Magno et al., 2019; Penny et al., 2021). The rat was placed in a glass cylinder, separated from the experimenters by a black curtain, and the number of rearings in the cylinder (Rc) during a 10-min period was recorded.

Retention of spatial memory was examined by the object location memory (OLM) test measuring the rat’s preference for an object in a novel versus familiar location (Gerstein et al., 2013). OLM was tested in the open field for 3 consecutive days. On day 1, each rat underwent two rounds of 5-min habituation in an empty arena. On day 2, the rat was allowed to explore two identical objects (LEGO blocks) placed in two corners of the arena (locations A and B) for a 10-min period. On day 3, in another 10-min session, rats were presented with the same location of one object (location A) and a novel location of the other object (location C). Frequencies of entry (FE) in the zone of the object at locations A, B, and C were recorded, and the frequency of entry ratios (FER) FEB/FEA and FEC /FEA were counted as discrimination factors between the two locations.

Sensorimotor function was assessed by the adhesive removal test (ART) (Komotar et al., 2007; Bouet et al., 2009). The test was performed in an empty transparent cage for 5 consecutive days. After 1 min of habituation, the rat was picked up by one experimenter, while the other attached an adhesive (1 cm^2^ of collage paper, sticky on one side) on its forepaws and returned it to the cage. The latency time to the beginning of removal (time-to-contact) and the time needed to remove the adhesive from the paws (time-to–remove) were recorded on the 1st day (initial response) and on the 5th day (after training). Average values for the left and right paw were used as the time-to-contact (TC in s) and time-to-remove (TR in s) which implied, respectively, paw and mouth sensitivity (time-to-contact) and dexterity (time-to-remove).

### 2.3 Tissue collection and homogenization

The tissue was collected from the subgroup of rats exposed to the initial set of behavioral tests. On P50, 16 rats (eight female rats and eight male rats), from the group exposed to hypoxia, and 15 rats (eight female rats and seven male rats), from the control group, were sacrificed in a randomized order. After administering isoflurane-provoked anesthesia (Abbott), the animal was decapitated, and the brain was quickly removed from the skull and dissected on a cold plate. The brain was cut in the midsagittal plane, and the hippocampus and thalamus were sampled separately, while mesencephalon and pons were isolated as a united sample (Supplementary Figure 1). Each collected sample was placed in a microtube and briefly weighed. The midbrain/pons tissue was kept on dry ice until homogenized with an ultrasonic homogenizer (Bandelin Sonopuls, Germany) in five volumes of deproteinizing solution (0.01 N HCl, 1 mM EDTA, 4 mM Na2S2O5) and stored at −20°C for the monoamine concentrations measurement. The thalamic and hippocampal tissue was kept in liquid nitrogen until disrupted and homogenized with the ultrasonic homogenizer in 12 volumes of lysis solution (Demeditec Diagnostics GmbH, Germany) and stored at −80°C for RNA isolation. To obtain the optimal amount of tissue for the isolation protocol, the thalamus was taken from one (left) hemisphere and the hippocampus from both hemispheres.

### 2.4 ELISA

Tissue homogenates were thawed, centrifuged at 24,000 × *g* and 4°C for 20 min, and an aliquot of the clear supernatant was used for the measurements of dopamine (DA), noradrenaline (NA), and adrenaline (ADR) concentrations using the 3-CAT Research ELISA (Demeditec Diagnostics GmbH, Germany) according to the kit instructions. After determining the optimal sample concentration for the reliable measurements of both DA and NA (ADR concentration in an aliquot was too low), samples were assayed in duplicates in a single assay (one for DA and one for NA). Intra-assay coefficients of variation, calculated from replicate standard curve vials, were 3.0% for DA and 4.2% for NA. A calibration curve was drawn based on the absorbance measured at 450 nm on a microplate reader (Bio-Rad, Germany) and known concentrations of the standard solutions. The concentration values of samples were obtained by interpolating them onto the calibration curve, using four-parameter non-linear regression curve fitting. The results were expressed in pg of DA/NA per mg of the wet brain tissue.

### 2.5 RNA isolation and qPCR

Total RNA was isolated using the phenol-free RNAqueous-4PCR kit (Ambion, Inc., Austin, TX, United States), and genomic DNA was removed, according to the manufacturer’s instructions. RNA concentration and quality were measured in a spectrophotometer (Biochrome) and assessed through agarose gel electrophoresis. From 1 μg of total RNA, mRNA was reversely transcribed using MuLV reverse transcriptase (Applied Biosystems, Foster City, CA, United States) and oligo dT primers (Applied Biosystems, Foster City, CA, United States), following the manufacturer’s instructions in a total volume of 20 μL. The performance of the reverse transcription was assessed through PCR using positive intron-spanning primers provided in the isolation kit. cDNA was stored at −20°C until further processing.

Data for five genes of interest (GOI)—*Drd1*, *Drd2*, *Ppp1r1b*, *Prkar2a,* and *Ppp1r1c—*and two endogenous references (ER)—*Actb* and *Hprt1—*are listed in Supplementary Table 1. To determine starting cDNA concentrations and to test the efficiency of amplification for all genes (efficiency between 90 and 110% is considered acceptable), 5-fold dilutions of pooled cDNA were used. The relative expression of genes was assessed through qPCR using the TaqMan gene expression master mix (Applied Biosystems, Foster City, CA, United States) according to the manufacturer’s instructions. The final volume of 20 μL contained 10 μL of master mix, 1 μL of the primers and probes for the reference gene (VIC labeled, primer-limited, Applied Biosystems) or 1 μL of the primers and probes for the gene of interest (FAM labeled, Applied Biosystems), 7 μL of nuclease-free H_2_O, and 2 μL of cDNA in the range of 50–60 ng per reaction. Samples were run in triplicates in singleplex reactions (for each sample, one GOI and both ERs were run on the same plate). One sample was randomly chosen as a calibrator and was loaded on each plate to correct experimental differences among consecutive PCR runs. The qPCR setup in the qTower3 PCR System (Analytik Jena, Germany) was 2 min at 50°C, 10 min at 95°C, followed by 40 cycles of 95°C for 15 s and 60°C for 60 s. The amplification results were analyzed with qPCRsoft 4.0 software (Analytik Jena, Germany). By using the equation 2^-ΔΔCt^, threshold cycles (Ct) of GOI and ERs were normalized to the corresponding threshold cycles of a calibrator, and the levels of GOI mRNAs were expressed relative to an average of the two endogenous references, i.e., in arbitrary units (AU).

### 2.6 Statistical analyses

Statistical analyses were performed using Prism8 (GraphPad Software, Inc., La Jolla, CA, United States) and JMP 11.2 (SAS Institute Inc., Cary, NC, United States). The normality of distribution was assessed by the Kolmogorov–Smirnov test. Values differing more than two standard deviations from the mean were considered outliers and were excluded from the statistical analyses if they did not comply with methodological standards (degrees of freedom indicating the analyzed number of samples for each test are listed in Supplementary Tables 2–4). The univariate split-plot approach was used to analyze CCtm, FER, TC, and TR by repeated measure ANOVA, with hypoxia and sex representing between-subject variables, and testing day/location representing within-subject variables. Kenward–Roger first-order approximation was used to calculate the degree of freedom of the denominator in case of the missing values. An independent two-way ANOVA was used, on original or transformed values, to check for the influence of hypoxia, sex, and their interaction on all other behavioral parameters as well as on catecholamine concentrations and relative GOI expression. If a significant effect was revealed, Tukey’s honest significance test was used for post-hoc analyses. Correlation between the parameters was calculated using Pearson or Spearman correlation coefficient, depending on the normality of distribution. A *p* value of <0.05 (two-tailed) was considered to be statistically significant. Values in the text were expressed as mean ± standard error of the mean (SEM).

## 3 Results

### 3.1 Behavioral alterations

**Figure 1:**
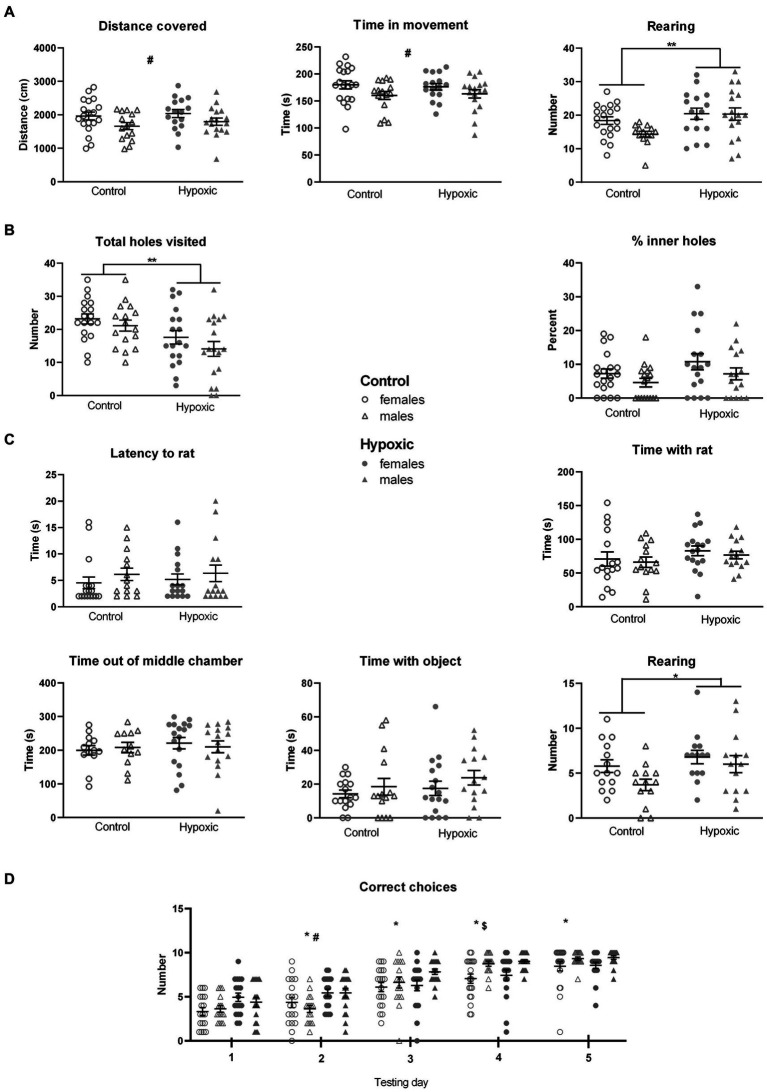
Behavior of 34 control (18 female rats and 16 male rats) and 36 hypoxic (18 female rats and 18 male rats) animals, at adolescent age, measured in **(A)** Open field test. Two-way ANOVA revealed significant effects of sex on distance covered and time in movement (^#^*p* < 0.05) and significant effects of treatment on the number of rearings (^**^p < 0.01). **(B)** Hole-board test. Two-way ANOVA revealed a significant influence of treatment on the total number of visited holes (^**^p < 0.01). **(C)** Social choice test. The only significant effect revealed by two-way ANOVA was the influence of treatment on the number of rearings (^*^p < 0.05). **(D)** *T*-maze test. Three-way repeated measure ANOVA revealed significant influences of treatment, testing day, sex × day interaction, and indicative influence of treatment × day interaction on the number of correct choices. Tukey’s honest significance *post hoc* test revealed significant differences among each testing day (^*^), between control and hypoxic animals on the second testing day (^#^), and between female and male rats on the fourth testing day (^$^). Results are shown as mean ± standard error.

The results of the initial set of behavioral tests are displayed in Figure 1, and all numerical values and statistical parameters are shown in Supplementary Table 2.

The perinatal hypoxia did not have a significant effect on locomotor activity, measured as distance covered (*F*_1,63_ = 0.00, *p* = 0.96) and time in movement (*F*_1,63_ = 0.83, *p* = 0.37) in an open field test (Figure 1A). On the other hand, this was the only aspect of behavior with significant sex influence, with female rats being more prone to horizontal movement than male rats (*F*_1,63_ = 5.74, *p* = 0.02 for DC, and *F*_1,63_ = 4.97, *p* = 0.03 for TM). A significant effect of hypoxia was observed in exploratory rearing. Compared to the controls, the affected rats displayed a significantly higher number of rearings when placed in a novel space (*F*_1,62_ = 7.60, *p* = 0.008).

The rats subjected perinatally to mild NH displayed significantly decreased novel object exploration tendency, measured as a reduced number of holes visited in a hole-board test (*F*_1,66_ = 10.8, *p* = 0.002), while the anxiety-like behavior, measured as a percentage of inner holes visited in a hole board, was not significantly affected by hypoxia (*F*_1,66_ = 3.23, *p* = 0.08; Figure 1B).

In a social choice test (Figure 1C), experimental groups did not differ with respect to sociability, measured as latency to approach the conspecific (*F*_1,63_ = 0.20, *p* = 0.66) and as the time spent in their exploration in a social choice test (*F*_1,59_ = 2.57, *p* = 0.11). Time spent out of the familiar middle chamber, representing a measure of anxiety-like behavior, and the time spent in an object exploration were not significantly affected by mild NH (*F*_1,63_ = 1.98, *p* = 0.17, and *F*_1,59_ = 1.03, *p* = 0.31, respectively). On the other hand, a tendency for novel space exploration, reflected in the number of rearings, was again significantly higher in the hypoxia-exposed group (*F*_1,54_ = 4.52, *p* = 0.04).

Reward-motivated spatial learning, tested as a number of correctly chosen T-maze arms containing food, during the 5 consecutive days (Figure 1D), was significantly affected by the perinatal hypoxia (*F*_1,66_ = 6.95, *p* = 0.01) and testing day (*F*_4,264_ = 155, *p* < 0.0001). Sex x day interaction was also significant (*F*_4,264_ = 5.14, *p* = 0.0005), while the *p* value for the treatment × day interaction was close to the threshold of 0.05 (*F*_1,63_ = 2.31, *p* = 0.06). The integral group of animals significantly improved their performance during the consecutive days. Interestingly, hypoxic animals had a higher number of correct choices than control animals during the first 2 days of testing and then reached similar values as the testing progressed. On the other hand, male rats had similar numbers of correct choices as female rats during the first 3 days of testing but outperformed the female rats toward the end of testing.

Sex × treatment interaction did not significantly affect any of the measured parameter, indicating similar consequences of the perinatal hypoxia in both sexes.

#### 3.1.1 Functional aspects of altered exploratory rearing

Since increased exploratory rearing emerged as the most prominent behavioral alteration in hypoxic animals, we further examined possible alterations in its functional aspects—spontaneous locomotion, somatosensory processing, and spatial learning. All numerical values and statistical parameters are shown in Supplementary Table 3.

**Figure 2:**
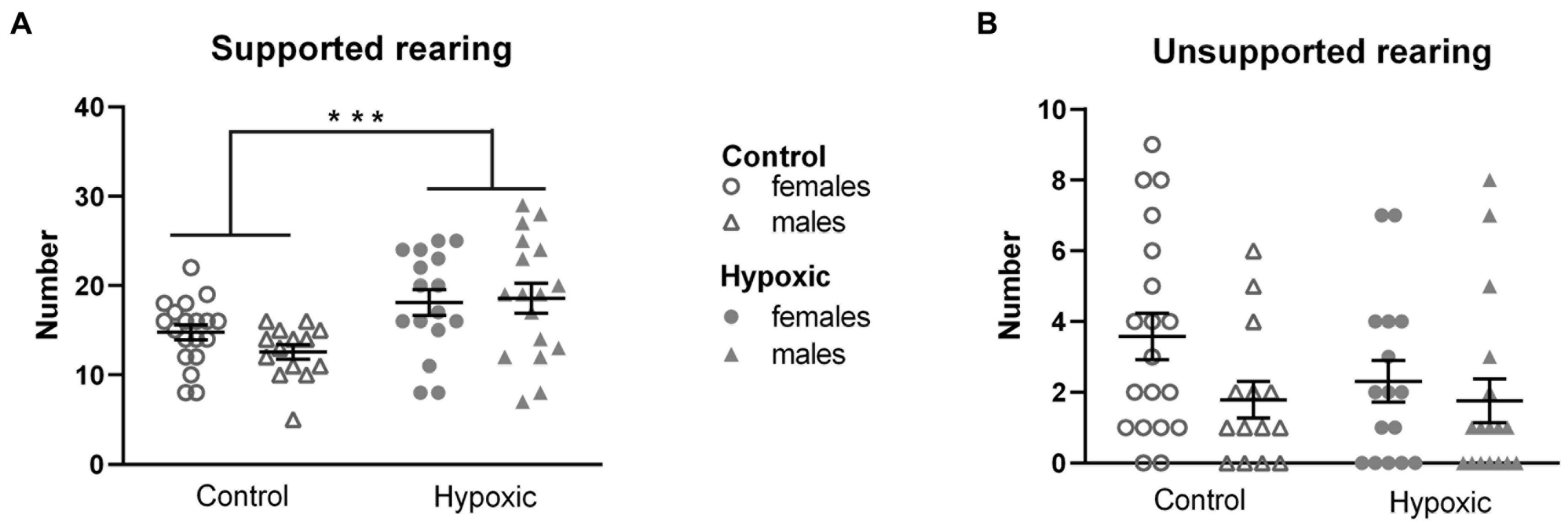
Rearing in open field of 34 control (18 females, 16 males) and 36 hypoxic (18 females, 18 males) rats divided into **(A)** supported rearing and **(B)** unsupported rearing. Two-way ANOVA revealed significant influence of treatment on SR (^***^p < 0.001). Results are shown as mean ± standard error.

**Figure 3:**
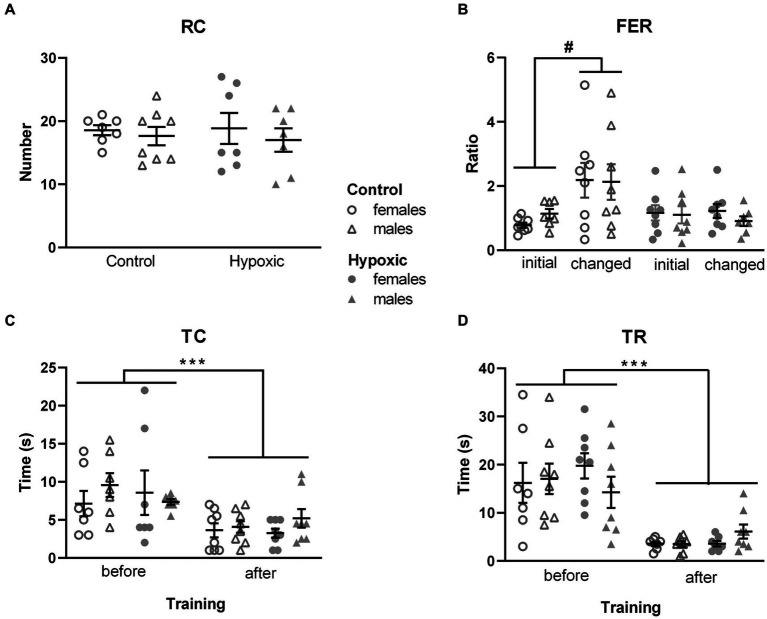
Behavior of 16 control (8F, 8 M) and 16 hypoxic (8F, 8 M) animals in additional behavioral tests intended to reveal the origin of increased rearing. **(A)** Spontaneous vertical locomotion was examined in a cylinder test as the number of rearings, RC. **(B)** Spatial memory was examined in the object location memory test as a ratio between frequencies of entry (FER) into the two object zones. After three-way repeated measure ANOVA showed significant effects of treatment x location interaction, Tukey’s honest significance *post hoc* test revealed significant differences in the object location discrimination in control but not in hypoxic rats (^#^*p* < 0.05). **(C)** Time to contact the adhesive (TC) and **(D)** time to remove the adhesive (TR) attached to the front paws were used to examine sensory-motor function in the adhesive removal test. Three-way repeated measure ANOVA revealed only significant effects of training on both TC and TR (^***^p < 0.0001).

Dividing the total number of rearings into unsupported (UR) and supported (SR) revealed that approximately 85% of all rearings were SR and only approximately 15% were UR (Figure 2). Accordingly, a significant effect of mild NH was observed only on supported rearing (*F*_1,62_ = 13.3, *p* = 0.0005, and *F*_1,62_ = 1.61, *p* = 0.21, respectively). We therefore measured the number of supported rearings in the cylinder test to check whether the hypoxic animals display a general increase in vertical locomotion when there is no open space to explore. Hypoxia did not significantly affect spontaneous upright movement measured as the number of supported rearings in a cylinder (*F*_1,25_ = 0.01, *p* = 0.92; Figure 3A).

In our study, perinatal hypoxic incident significantly impaired spatial memory (Figure 3B), which was reflected in significant hypoxia × location interaction (*F*_1,28_ = 7.99, *p* = 0.009). For both hypoxic and control animals, the frequency of entries into the zones of the two objects placed in an open field was similar in the initial test (FER = 0.96 for control and FER = 1.13 for hypoxic group). However, when one of the objects was displaced in the second test, control animals visited the object twice as many times as the object placed in the familiar location (FER = 2.15), while this shift did not occur in the hypoxic group (FER = 1.06).

As alterations in the novel object exploration after perinatal hypoxia indicated the possibility of altered somatosensory processing, we assessed the sensorimotor function in the adhesive removal test. Mild perinatal hypoxia did not affect sensory function, measured as the time to contact the adhesive attached to the front paws (*F*_1,27_ = 0.00, *p* = 0.96; Figure 3C), nor the dexterity, registered as the time to remove the adhesive (*F*_1,27_ = 0.22, *p* = 0.64; Figure 3D). Both parameters were influenced only by training (*F*_1,27_ = 17.4, *p* = 0.0003 for TC and *F*_1,27_ = 63.0, *p* < 0.0001 for TR) as all four subgroups were more efficient in performing the tasks after 3 training days.

None of the tested parameters was significantly affected either by sex or treatment × sex interaction.

### 3.2 Alterations in catecholamine neurotransmission pathways

**Figure 4:**
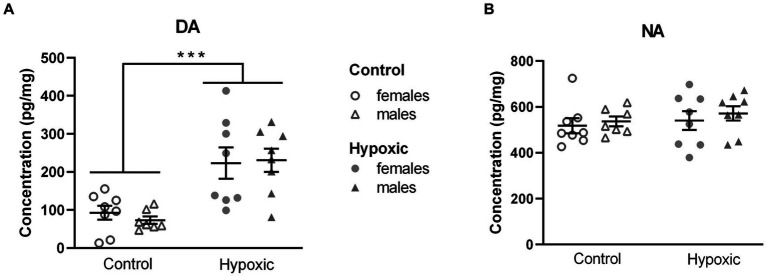
Midbrain/pons concentration of **(A)** dopamine (DA) and **(B)** noradrenaline (NA), in pg/mg, in 15 control (8F, 7 M) and 16 hypoxic (8F, 8 M) animals. Results are shown as mean ± standard error. ^***^p < 0.0001, effect of treatment by two-way ANOVA.

The subsequent question that arose was whether the found alterations in exploratory rearing were catecholamine-mediated. We therefore measured NA and DA concentrations in the region abundant with dopaminergic neurons (Supplementary Figure 1) in a subgroup of animals (Figure 4). All numerical values and statistical parameters for the catecholamine-related parameters are shown in Supplementary Table 4. While hypoxia did not significantly affect NA concentrations (*F*_1,27_ = 0.76, *p* = 0.30), mean DA concentrations were approximately three times higher in the hypoxic than in the control group (*F*_1,27_ = 25.8, *p* < 0.0001). Sex and sex × hypoxia interaction did not significantly affect catecholamine concentrations. Interestingly, statistically significant positive correlations were observed between midbrain/pons DA concentrations and number of exploratory rearings (*r* = 0.466, *p* = 0.008) and, more specifically, supported rearings (*r* = 0.474, *p* = 0.007).

**Figure 5:**
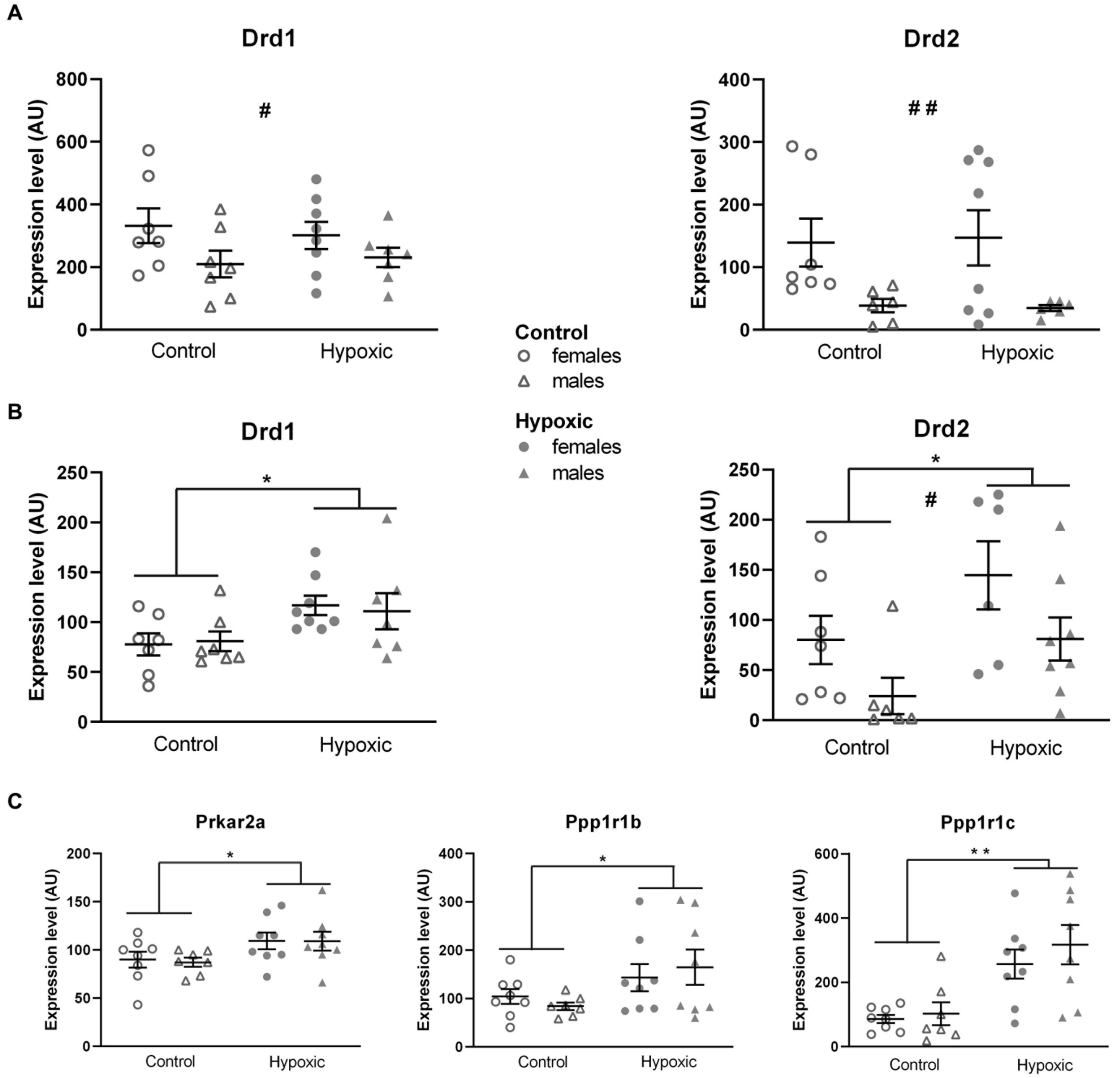
Relative expression of the genes for dopamine receptors D1 (*Drd1*) and D2 (*Drd2*) in the hippocampus **(A)** and thalamus **(B)** and for D1 and D2 downstream signalization molecules: regulatory subunit of protein kinase A, PKArs (*Prkar2a*) dopamine- and cAMP-regulated phosphoprotein, DARP-32 (*Ppp1r1b*), and inhibitor-5 of protein phosphatase 1, IPP5 (*Ppp1r1c*) in the thalamus **(C)**, of 15 control (8F, 7 M) and 16 hypoxic (8F, 8 M) animals. mRNA levels are expressed in arbitrary units (AU), i.e., normalized to a calibrator and relative to an average of the two endogenous references, as 2^-ΔΔCt^ × 100. Results are shown as mean ± standard error. ^*^p < 0.05 and ^**^p < 0.001, significant influence of treatment; ^#^*p* < 0.05 and ^##^*p* < 0.001, significant effect of sex; two-way ANOVA.

Considering that our hypoxic animals displayed spatial memory deficits, we further explored the influence of increased midbrain/pons DA content on the DA neurotransmission in two regions involved in spatial processing—the thalamus and hippocampus. Possible alterations in DA signaling were determined by relative expression analysis of the genes for D1 and D2 receptors (*Drd1*, *Drd2*; Figure 5). While hypoxia did not significantly affect the relative expression of *Drd1* (*F*_1,25_ = 0.01, *p* = 0.91) and *Drd2* (*F*_1,23_ = 0.05, *p* = 0.82) in the hippocampus (Figure 5A), the expression of both genes was significantly upregulated (*F*_1,25_ = 7.59, *p* = 0.01 and *F*_1,23_ = 7.44, *p* = 0.01, respectively) in the thalamus (Figure 5B). To confirm altered DA signaling in the thalamus, we further analyzed the relative expression of the genes for the receptors’ downstream targets: the regulatory subunit of protein kinase A, PKArs (*Prkar2a*), the dopamine- and cAMP-regulated phosphoprotein, DARPP-32 (*Ppp1r1b*), and inhibitor-5 of protein phosphatase 1, IPP5 (*Ppp1r1c*) (Figure 5C). Changes in the expression of all three genes pointed in the same direction, with a significant increase in the hypoxic group compared to the control group (*F*_1,27_ = 6.17, *p* = 0.02 for *Prkar2a*, *F*_1,27_ = 5.56, *p* = 0.03 for *Ppp1r1b*, and *F*_1,63_ = 20.0, *p* = 0.0001 for *Ppp1r1c*). Sex had a significant effect on the expression of *Drd1* in the hippocampus (*F*_1,25_ = 4.74, *p* = 0.04) and the expression of *Drd2* in the hippocampus (*F*_1,23_ = 9.51, *p* = 0.005) and thalamus (*F*_1,23_ = 6.95, p = 0.02), with higher expression in female rats. The expression of other genes was not affected by sex. Hypoxia × sex interaction did not significantly affect the relative expression of the mentioned genes.

## 4 Discussion

We have studied behavioral alterations, catecholamine content, and gene expression involved in dopaminergic signaling in adolescent rats that underwent mild normobaric hypoxia at P1. The study elucidates a high vulnerability of the immature dopaminergic neurotransmitter system. An increase in the midbrain/pons DA content and elevated expression of genes for the thalamic DA receptors and downstream signaling proteins was accompanied by deviated exploratory rearing behavior with impaired spatial learning.

### 4.1 Milder behavioral alterations were found in rats with HBI induced in normobaric compared to hypobaric conditions

To examine the behavioral effects of mild normobaric hypoxia and compare it to those of moderate hypobaric hypoxia observed in our previous study (Trnski et al., 2022), exploratory behavior, sociability, and cognitive performance, reported in literature as vulnerable to perinatal hypoxic events, were studied on a large group of adolescent rats in a set of behavioral tests.

Perinatal exposure to hypoxia has been frequently reported to affect locomotor activity and exploratory tendency, as the aspects of novel space exploration, without affecting anxiety. Increased ambulation in an open field was observed in juvenile rats exposed to intermittent hypoxia at P7-11 (Decker et al., 2005; Yamada et al., 2014), chronic NH at P0-21 (Mikati et al., 2005), as well as anoxia at P2 (Rogalska et al., 2004) and P4 (Shimomura and Ohta, 1988). A significant increase in both ambulation and rearing was reported in pre-pubertal rats after P2 NH (Ordyan et al., 2017) and juvenile rats after P0 anoxia (Speiser et al., 1983; Dell'Anna et al., 1991; Iuvone et al., 1996). No differences in open field and/or zero-maze thigmotaxis were observed in juvenile and/or adult rats after an invasive HI at P7 (Arteaga et al., 2015) or after a severe P0 asphyxia (Galeano et al., 2011; Herrera et al., 2018; Vázquez-Borsetti et al., 2019). In our laboratory setup, previously performed HH also evoked a significant increase in locomotor activity and exploratory tendency without affecting the anxiety level. In contrast, the exposure to NH in this study significantly affected only exploratory rearing both in the open field and social choice apparatus. Assuming that NH represents a milder insult than HH, we can conclude that rearing might be the aspect of novel space exploration that is most vulnerable to a hypoxic event.

An increase in novel object exploration after perinatal exposure to hypoxia has been reported as an increased frequency of head-dipping in the hole-board after a P7 HI (Arteaga et al., 2015) or as an increased time of object exploration after P1 anoxia (Laviola et al., 2004). HH in our previous study affected novel object exploration very mildly—only in a hole board and only in male rats. As opposed to previously reported findings (Trnski et al., 2022), the hole-board test in this study revealed a significant decrease in the number of visited holes in the entire group exposed to NH. This finding may be explained by an observation that hypoxic rats frequently dipped their head in a hole only partially, not fulfilling the criterion for scoring. Regardless of the underlying cause, which may include increased cautiousness or altered sensory processing, the hole-board test did not suggest an increase in novel object exploration. The lack of influence on the time spent exploring an object in the social choice test points in the same direction.

Sociability seems to be less vulnerable to milder hypoxic events. In adolescent rats, P12 ischemia did not alter social interaction (Tejkalová et al., 2007), P0 asphyxia selectively decreased play soliciting while leaving other aspects of social interaction intact (Vázquez-Borsetti et al., 2019), and only HI at P7 significantly lowered social preference (Ji et al., 2015) and at P0 increased social avoidance (Driscoll et al., 2018). In our studies, previous HH reduced sociability only in female rats, while the current NH did not affect sociability measured either as a latency to approach or as a time to explore a conspecific in a social choice test.

Cognitive alterations induced by perinatal hypoxia/anoxia have been described as impairments in working or reference memory in various spatial navigation tasks (Dell'Anna et al., 1991; Buwalda et al., 1995; Balduini et al., 2000; Decker et al., 2003; Mikati et al., 2005; Raveendran and Skaria, 2013). In our previous study, HH induced transient sex-specific learning impairment, with a significantly decreased number of correct choices in a T-maze in juvenile hypoxic male rats compared to the control male rats. On the contrary, NH in this study did not seem to impair T-maze performance. It is possible that our experimental conditions were mild compared to the moderate conditions used in the abovementioned studies. Similarly, Mikati et al. (2005) showed that acute hypoxia (4% O2) at P10, but not mild chronic hypoxia (10% O2) at P0–P21, decreased performance in the Morris water maze. Surprisingly, our hypoxic animals outperformed control rats during the first 2 days of testing but then leveled up with the controls for the rest of the testing period. This phenomenon might be explained by increased motivation for a reward in the hypoxic group that improved working memory in the early testing phase and compensated for a potential deficit in reference memory as the testing continued. Another possible explanation points to the opposite—while the spontaneous alteration of the T-maze arms (Richman et al., 1986) was suppressed in the control group by repeated presence of the reward in the same arm, the hypoxic group might not have associated the correct arm choice with the reward (Win/Stay strategy) in the early phase of testing and kept displaying spontaneous alteration. Indeed, within a 10-trial session during the first 2 days of testing, hypoxic animals would always walk to either arm of the maze (being correct or false), while the control animals would stop in many trials at the branching point and head-scanned until the testing time of a trial was over, rendering the outcome a false choice. The latter behavior was observed in our previous studies, and increasing the trial time did not result in an arm choice.

As opposed to our previous study in which exposure to hypobaric HBI differentially affected locomotor behavior and spatial learning in a sex-dependent manner, normobaric HBI in this study seemed to affect male and female pups equally, indicating that the sex-specific adverse effects of hypoxia may become detectable only after a more serious insult. Statistically significant sex-related differences in locomotor activity and spatial learning were noticed only generally between female and male rats regardless of treatment. This is in accord with published data reporting a higher level of locomotion in female rodents and a better spatial learning in male rodents (Belviranli et al., 2012; Chen et al., 2020), presumably underpinned by anatomical differences in the corresponding neuronal circuits induced by the neonatal testosterone surge in male rats (Clarkson and Herbison, 2016).

### 4.2 The impaired spatial learning might explain increased exploratory rearing

The possible functional relevance of altered exploratory rearing was examined by additional behavioral tests (CT, ART, and OLM).

Rearing in an open field is a measure of novel space exploration and represents a joint outcome of several separate aspects—inherent locomotor activity, exploratory tendency, and anxiety level. When a rat is placed in a narrow cylinder, there is no possibility of ambulation (hence, no space to explore and map), and no distinction between open space and walls, so rearing in the cylinder is considered to be the only result of inherent locomotor activity. The number of spontaneous rearings in CT was not altered in the hypoxic group, showing that, in our study, the inherent vertical locomotor activity, similar to the horizontal one, has not been affected by NH.

The lack of significant differences in ART between control and hypoxic animals indicated that NH did not affect sensory-motor processing. Similarly, no change in ART performance in adult rats was reported after P11 asphyxia (Gailus et al., 2021), P3 HI (Sanches et al., 2017), and adult NH (Yan et al., 2011). On the other hand, unilateral severe P7 HI was reported to increase adhesive removal time from both the affected and unaffected paw (Lowe et al., 2017). ART also revealed that both experimental groups significantly improved performance after 2 days of training, indicating that implicit learning in our hypoxic animals remained intact.

In the OLM test, hypoxic rats did not prefer exploring an object moved to a novel location, which was clearly seen in the control group. Several factors, such as locomotor activity, motivation, or anxiety level might have affected the performance of the hypoxic group in the OLM test. However, the initial set of behavioral tests did not show hypoxia-related alterations in the locomotor activity and anxiety level. In addition, the influence of the mentioned factors would have been reflected in the total frequencies of entry (FEA + FEB and FEA + FEC), but they did not differ between the groups (65 vs. 61 total entries in the first test and 66 vs. 68 total entries in the second test for control and hypoxic rats, respectively). Therefore, we consider the lack of preference for an object moved to a new location to be a sign of impairment in spatial memory. This result is in accordance with findings of decreased exploratory preference for an object in a non-familiar location after neonatal hipoxia-ischemia in P7 rats (Tata et al., 2015) and P10 mice (Spahic et al., 2022). It is also consistent with reports of impaired habituation memory in juvenile rats in the open field test (Sab et al., 2013) and an inability to discriminate between the new and familiar object in the novel object recognition memory test (Cunha-Rodrigues et al., 2018) after prenatal hipoxia-ischemia, suggesting that the exposure to NH affected retention of spatial memory 24 h after the initial test. Slightly different paradigms of testing may explain the distinction between the results obtained in OLM and T-maze tests. Unlike the T-maze test, which combines working memory, reference memory, and reward-based motivation, OLM uses natural rodent preference for novelty and does not depend on the retention of a rule or responsivity to reward (Ennaceur et al., 1997), representing a more sensitive measure of impairments in spatial learning.

Although rearing has been reported as an aspect of exploration affected by various hypoxic events, none of the studies mentioned in Section 4.1 distinguished between SR and UR. While UR is agreed upon to represent a hippocampus-mediated spatial mapping behavior, the function of SR is yet to be unraveled and has been suggested to represent the noradrenaline-mediated escape attempt, dopamine-mediated general increase in locomotion, or way of acquiring somatosensory space perception (Lever et al., 2006). In our study, rats reared predominantly by leaning against the wall, while unsupported rearings were scarce in both groups. Considering that rats are prone to thigmotaxic behavior, characterized by avoiding the center of open field apparatus, such a high portion of SR in the total number of rearings is not unexpected. Since the number of UR was too low for reliable statistical comparison, we can only claim a statistically significant effect of hypoxia on SR, which corresponded with the level of retention of spatial memory, but not with the inherent locomotor activity and sensory-motor processing.

### 4.3 Increased midbrain/pons DA content and altered thalamic DA signaling are observed in hypoxic rats

We further examined a possible catecholaminergic background of the increased exploratory rearing by measuring the midbrain/pons NA and DA content. The lack of differences in NA levels between the hypoxic and control groups diminished the possibility of explaining the increased SR as the NA-mediated escape attempt. On the other hand, DA levels increased threefold in hypoxic compared to control animals and significantly correlated with a number of exploratory rearings, pointing at a dopaminergic mediation of this behavior. Our results align with those obtained by Seidler and Slotkin (1990), who reported increased DA but not NA content after P1 hypoxia. An increase in the midbrain DA content and the number of DA-containing neuronal cell bodies was found after chronic intermittent hypoxia (Raghuraman et al., 2009) and after neonatal asphyxia (Bjelke et al., 1991; Chen et al., 1997). Alterations of the midbrain DA homeostasis after perinatal hypoxia were also observed in the human brain post-mortem (Pagida et al., 2013) and in the mesencephalic cell culture following exposure to non-damaging hypoxia during *in vitro* days 1–3 (Gross et al., 1999).

Hypoxia-induced rise in the midbrain/pons DA content could be expected to affect dopaminergic neurotransmission in the target regions. Alterations in DA signaling have been predominantly studied in the main midbrain DA targets—basal ganglia, for which an increase in dopaminergic innervation (Burke et al., 1991), extracellular DA concentration (Akiyama et al., 1991), and expression of DA-regulating elements (Boksa et al., 2002; Decker et al., 2003) has been reported, while the target regions involved in spatial learning have been far less explored. The hippocampus, already established as a key component of spatial processing (Bird and Burgess, 2008), receives the DA input from dopaminergic neurons in the VTA and substantia nigra (Gasbarri et al., 1994) as well as from noradrenergic neurons in the locus coeruleus (LC) (Kempadoo et al., 2016). The thalamus, particularly its anterior part containing head direction cells and dense interconnections with the limbic cortex (in which our previous study of HBI under hypobaric conditions revealed structural changes) and hippocampal formation, has been recently recognized as a critical mediator of spatial orientation (Jankowski et al., 2013).

Although recent optogenetic studies showed that midbrain DA communication with the hippocampus modulates the acquisition of long-term memory (McNamara et al., 2014; Rosen et al., 2015), this study did not indicate hippocampal changes in DA signalization, measured as relative mRNA levels for D1 and D2 receptors. Since we used RNA isolated from the whole hippocampus, discrete changes in *Drd1* and/or *Drd2* expression in specific regions could have gone unnoticed and should not be ruled out. Nevertheless, our current results do not support the involvement of dopaminergic signaling in the hippocampus in the hypoxia-induced memory deficit. Indeed, memory impairments in animal models, induced by H and HI, have been linked to the long-term hippocampal alterations in excitatory/inhibitory balance. An increase in extracellular acetyl-choline and a decrease in extracellular glutamate concentrations have been reported in adolescent rats after P7 hypoxia (López-Pérez et al., 2012). Additionally, an increase in the extracellular GABA level has been observed in adult rats after P10 hypoxia (Pozdnyakova et al., 2011), along with a higher rate of GABA synthesis in young rats after prenatal HI (Cunha-Rodrigues et al., 2018).

On the other hand, we are the first to report that dopaminergic alterations in the thalamus accompany cognitive impairment following a mild hypoxic event. Altered dopaminergic signaling in the thalamus, indicated by the significant increase in *Drd1* and *Drd2* expression, was confirmed by the corresponding increase in mRNA levels for the main downstream proteins in the D1/D2 signaling pathway: the regulatory subunit of PKA and the two inhibitors of PP-1: DARPP-32 and IPP5. An increased abundance of DA-pathway mRNAs in the thalamic tissue may represent the consequence of a long-lasting upregulation of dopaminergic pathway genes in the thalamic neurons following neonatal hypoxia. The expression of D1/D2 downstream elements after hypoxia has been scarcely investigated. A 2-fold increase in DARPP-32 abundance was noted in rat cortex following P7 hypoxia (Hu et al., 2006), but no reports of the long-term effects on expression could be found. Increased *Prkar2a* expression, related to a low-oxygen/high-altitude adaptation, was observed in the hearts of Tibetan pigs in comparison to low-land pigs (Zhang et al., 2019), while the expression of *Ppp1r1c* has been studied only in tumor hypoxia. Alternatively, increased abundance of DA-pathway mRNAs may result from structural changes induced by an increased dopaminergic innervation of the thalamus. The latter possibility is supported by the findings of a long-lasting increase in DA release sites in the rat striatum and nucleus accumbens after neonatal exposure to HI (Burke et al., 1991) as well as in the mouse striatum after prenatal exposure to hypoxia (Brandon et al., 2022).

By controlling the state of phosphorylation of a variety of downstream physiological effectors, the PKA/DARPP-32/PP-1 cascade was shown to regulate the functional state of neurons in the striatum (Greengard et al., 1998; Svenningsson et al., 2004), the activity of proteins involved in the MAPK signaling pathway in the prefrontal cortex (Nagai et al., 2007), and the activity of several transcription factors, including CREB, in the caudate putamen (Di Benedetto et al., 2007). It is, therefore, possible to presume that the hypoxia-induced alteration in DA signaling might have affected thalamic function and its communication with other brain regions involved in the mediation of exploratory behavior.

## 5 Conclusion

Mild perinatal HBI obtained under normobaric conditions presented in this study has generally affected similar behavioral aspects as previously presented HBI obtained under hypobaric conditions (exploration and cognition) but to a much lesser extent. This aligns with more severe physiological effects of HH than NH found in adult animals and humans (Savourey et al., 2003).

The set of initial behavioral tests pointed out exploratory rearing as the most prominent alteration induced by perinatal hypoxia regardless of hypo- or normobaric conditions. Our search for the functional aspect of rearing that contributed to the observed increase in this type of behavior identified lasting impairment of spatial learning, rather than impaired somatosensory processing or increased inherent vertical locomotion, in animals exposed to hypoxia.

Altered exploratory rearing was paralleled by a considerable increase of DA content in the midbrain/pons and by significant alterations in the thalamic dopaminergic signaling. The increased mRNA levels for DA receptors and their downstream elements likely resulted in increased levels of their protein products, which, depending on the regional D1 and D2 distribution, may have enhanced or attenuated short-term and long-term effects of DA in the thalamus. Altered DA signaling in the thalamus could have affected its communication with the target regions, including the hippocampus and cingulate cortex, indirectly contributing to the alterations in spatial processing.

We can conclude that NH is the least possible perinatal intervention on an animal model that can still induce measurable behavioral, neurochemical, and molecular alterations, and is therefore suitable for studying mild prenatal hypoxic events in humans. Further studies on our model, aimed at structural and molecular changes in particular thalamic nuclei as well as in the thalamocortical and thalamo-hippocampal projections, should give a closer insight into the role of mesothalamic and mesolimbic dopaminergic pathways in hypoxia, opening a possibility for studying new approaches to treat long-term behavioral outcomes after a hypoxic–ischemic insult.

## Data availability statement

The original contributions presented in the study are included in the article/Supplementary material, further inquiries can be directed to the corresponding authors.

## Ethics statement

The animal study was approved by the ethical committee of the University of Zagreb and national ethical and animal welfare bodies (EP231/2019; UP/I-322-01/19.01/75). The study was conducted in accordance with the local legislation and institutional requirements.

## Author contributions

BN: Investigation, Methodology, Visualization, Writing – review & editing. ST-L: Investigation, Methodology, Visualization, Writing – review & editing. KK: Investigation, Writing – review & editing. MD: Visualization, Writing – review & editing. IB: Formal Analysis, Writing – review & editing. DH: Conceptualization, Formal Analysis, Supervision, Writing – original draft. NJ-M: Conceptualization, Funding acquisition, Project administration, Supervision, Writing – review & editing.
