# Supplementary material for: Lasting mesothalamic dopamine imbalance and altered exploratory behavior in rats after a mild neonatal hypoxic event
Source: Front Integr Neurosci. 2024 Jan 17;17:1304338. doi: 10.3389/fnint.2023.1304338 (PMC10832065; doi:10.3389/fnint.2023.1304338)
Supplement: Supplementary file 1 [file Table_1.pdf]

Supplementary Table 1. Genes used in qPCR

Symbol	Full name	Description	TaqMan assay	Correlation coefficient	Slope	Amplification efficiency (%)
Actb	actin, beta		Rn00667869_m1	0,99	-3,59	90
Hprt1	hypoxanthine phosphoribosyltransferase 1		Rn01527840_m1	0,99	-3,52	92
Drd1	dopamine receptor D1		Rn03062203_s1	0,99	-3,54	92
Drd2	dopamine receptor D1		Rn00561126_m1	0,99	-3,39	97
Ppp1r1b	protein phosphatase 1, regulatory (inhibitor) subunit 1B	gene for dopamine- and cAMP-regulated phosphoprotein, Mr 32 kDa (DARPP-32)	Rn01452984_m1	0,97	-3,6	90
Prkar2a	protein kinase cAMP-dependent type II regulatory subunit alpha	gene for regulatory subunit of protein kinase A (PKArs)	Rn00709403_m1	0,97	-3,32	100
Ppp1r1c	protein phosphatase 1, regulatory (inhibitor) subunit 1C	inhibitor-5 of protein phosphatase 1 (IPP5)	Rn01513845_m1	0,96	-3,24	103

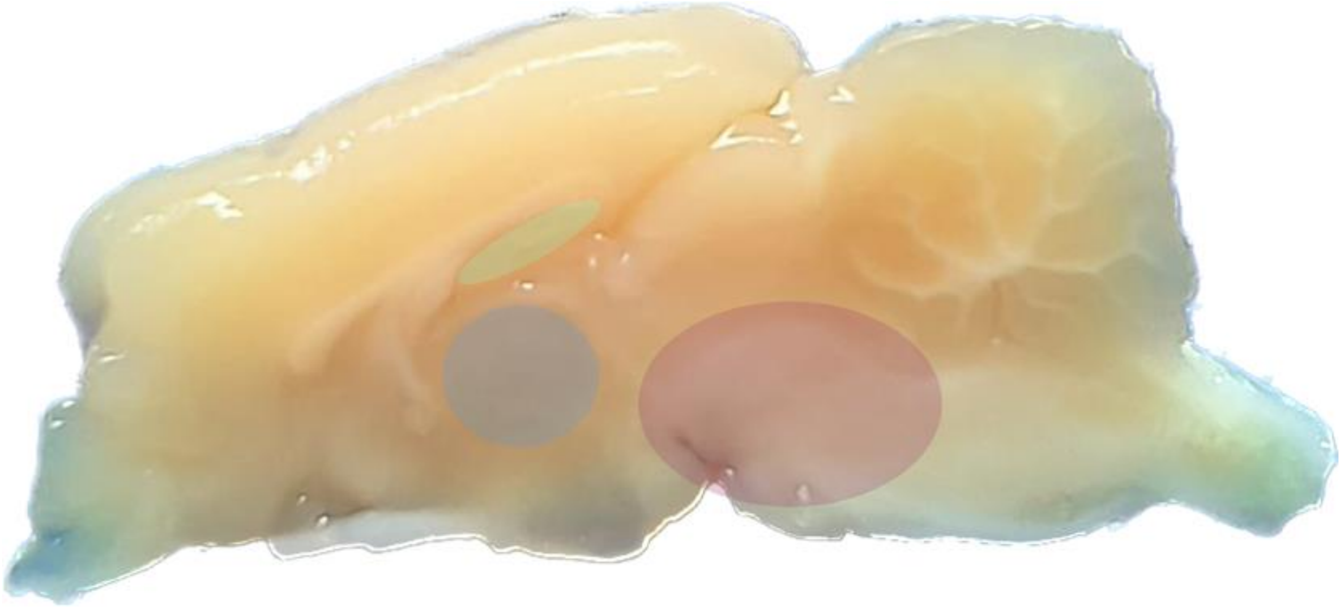

Supplementary Figure 1. The photography presents the rat brain at P50, cut in the mid-sagittal plane. Transparent demarcations indicate the brain regions: pink - mesencephalon and pons (used for measurements of dopamine and noradrenaline concentrations), green – hippocampus (used for measurements of mRNA levels of dopamine receptors), and blue – thalamus (used for measurements of mRNA levels of dopamine receptors and downstream proteins).

Supplementary Table 2. Values and statistics for parameters measured in the initial battery of behavioral tests.

Test	Parameter	Sex	VALUES (M ± SEM) Treatment control	hypoxic	Sex mean	STATISTICAL PARAMETERS Treatment	Sex	Treatment*Sex
Open field	DC	male	1662 ± 120	1796 ± 113	**1733 ± 82**	F(1,63)=0.002	F(1,63)=5.74	F(1,62)=0.07
		female	1965 ± 107	2039 ± 116	**1999 ± 79**	p=0.9625	**p=0.0196**	p=0.7964
		**Treatment mean**	**1831 ± 80**	**1914 ± 81**		η_p^2 =0.000	η_p^2 = 0.084	η_p^2 =0.001
	TM	male	160 ± 7.40	163 ± 7.67	**162 ± 5.31**	F(1,63)=0.83	F(1,63)=4.97	F(1,63)=0.20
		female	180 ± 7.65	176 ± 6.13	**178 ± 5.08**	p=0.3651	**p=0.0294**	p=0.6553
		**Treatment mean**	**171 ± 5.17**	**170 ± 5.22**		η_p^2 =0.013	η_p^2 = 0.073	η_p^2 =0.003
	R	male	14.4 ± 1.58	20.4 ± 1.43	**17.6 ± 1.07**	F(1,62)=7.60	F(1,62)=1.96	F(1,62)=1.80
		female	18.4 ± 1.35	20.4 ± 1.48	**19.3 ± 1.00**	**p=0.0076**	p=0.1664	p=0.1843
		**Treatment mean**	**16.7 ± 1.04**	**20.4 ± 1.03**		η_p^2 =0.109	η_p^2 = 0.031	η_p^2 =0.028
Hole board	THV	male	21.1 ± 1.74	14.1 ± 2.31	**17.4 ± 1.37**	F(1,66)=10.8	F(1,66)=2.10	F(1,66)=0.15
		female	23.2 ± 1.49	17.6 ± 2.16	**20.4 ± 1.33**	**p=0.0016**	p=0.1518	p=0.7040
		**Treatment mean**	**22.2 ± 1.37**	**15.9 ± 1.33**		η_p^2 =0.141	η_p^2 =0.031	η_p^2 =0.002
	%IN	male	4.59 ± 1.35	7.28 ± 1.72	**6.27 ± 1.31**	F(1,66)=3.23	F(1,66)=2.27	F(1,66)=0.03
		female	7.39 ± 1.41	10.8 ± 2.48	**8.91 ± 1.22**	p=0.0770	p=0.1373	p=0.8583
		**Treatment mean**	**6.16 ± 1.24**	**9.36 ± 1.29**		η_p^2 =0.047	η_p^2 =0.033	η_p^2 =0.000
Social choice	ToMC	male	208 ± 15.4	223 ± 15.2	**216 ± 11.4**	F(1.54)=1.98	F(1.54)=0.33	F(1.54)=0.23
		female	192 ± 16.3	221 ± 9.06	**208 ± 10.8**	p=0.1652	p=0.5676	p=0.6359
		**Treatment mean**	**200 ± 11.8**	**222 ± 10.4**		η_p^2 =0.029	η_p^2 =0.005	η_p^2 =0.003
	TO	male	18.5 ± 5.36	23.8 ± 4.29	**21.1 ± 2.91**	F(1,59)=1.03	F(1,59)=1.56	F(1,59)=0.10
		female	14.3 ± 2.26	17.5 ± 4.42	**16.2 ± 2.73**	p=0.3144	p=0.2164	p=0.7487
		**Treatment mean**	**16.5 ± 2.82**	**20.5 ± 2.82**		η_p^2 =0.017	η_p^2 =0.026	η_p^2 =0.002
	TR	male	61.7 ± 8.86	76.7 ± 5.11	**69.2 ± 5.87**	F(1,59)=2.57	F(1,59)=1.03	F(1,59)=0.047
		female	70.7 ± 8.33	82.8 ± 6.03	**77.4 ± 5.51**	p=0.1142	p=0.3136	p=0.7964
		**Treatment mean**	**67.1 ± 5.87**	**79.9 ± 5.69**		η_p^2 =0.042	η_p^2 =0.017	η_p^2 =0.001
	LtR	male	6.14 ± 1.23	6.33 ± 1.00	**6.26 ± 1.44**	F(1,59)=0.20	F(1,59)=0.23	F(1,59)=0.004
		female	4.53 ± 1.11	5.19 ± 1.19	**4.88 ± 1.61**	p=0.6560	p=0.6354	p=0.9530
		**Treatment mean**	**4.92 ± 1.95**	**5.89 ± 0.92**		η_p^2 =0.003	η_p^2 =0.004	η_p^2 =0.000
	R	male	3.69 ± 0.65	6.00 ± 0.66	**4.89 ± 0.55**	F(1,54)=4.52	F(1,54)=3.43	F(1,54)=0.71
		female	5.79 ± 0.73	6.79 ± 0.51	**6.29 ± 0.54**	**p=0.0383**	p=0.0699	p=0.4043
		**Treatment mean**	**4.78 ± 0.55**	**6.39 ± 0.54**		η_p^2 =0.077	η_p^2 =0.060	η_p^2 =0.013
T-maze		Day	Treatment					
	CC females	1	3.32 ± 0.41	4.94 ± 0.51		Sex	Treatment	Day
		2	4.31 ± 0.57	5.26 ± 0.45		F(1,66)=2.31	F(1,66)=6.95	F(4,264)=155
		3	6.11 ± 0.51	6.11 ± 0.61		p=0.1335	**p=0.0104**	**p<0.0001**
		4	7.05 ± 0.53	7.26 ± 0.61		η_p^2 =0.034	η_p^2 =0.095	η_p^2 =0.701
		5	8.47 ± 0.55	8.37 ± 0.39		Sex*Treatment	Sex*Day	Treatment*Day
	CC males	1	3.47 ± 0.39	4.33 ± 0.56		F(1,66)=0.01	F(4,264)=5.14	F(4,264)=2.31
		2	3.53 ± 0.41	5.28 ± 0.53		p=0.9332	**p=0.0005**	p=0.0582
		3	6.41 ± 0.65	7.50 ± 0.40		η_p^2 =0.000	η_p^2 =0.072	η_p^2 =0.034
		4	8.47 ± 0.40	8.67 ± 0.34		Sex*Treatment*Day		
		5	9.06 ± 0.32	9.17 ± 0.32		F(4,264)=0.96	p=0.4279	η_p^2 =0.014

DC-distance covered, TM- time in movement, R- number of rearings, THV-total holes visited, %IN- percentage of inner holes visited, ToMC-time out of middle chaimber, TO-time exploring an object, TR-time exploring a rat, LtR-latency to approach the rat, CC-correct choices. η_p^2 - partial eta squared , 0.01 indicating a small, 0.06 a medium, and 0.14 a large effect size (https://effect-size-calculator.herokuapp.com/).

Supplementary Table 3. Values and statistics for parameters measured in additional behavioral analyses

Test	Parameter		VALUES (M ± SEM)			STATISTICAL PARAMETERS		
			**control**	**hypoxic**				
**Rof**		**Sex**	**Treatment**		**Sex mean**	Treatment	Sex	Treatment*Sex
		male	13.0 ± 1.30	18.6 ± 1.23	**16.0 ± 0.89**	F(1,62)=13.3	F(1,62)=0.24	F(1,62)=0.75
	**SR**	female	14.7 ± 1.16	18.1 ± 1.26	**16.3 ± 0.86**	**p=0.0005**	p=0.6239	p=0.3895
		**Treatment mean**	**13.9 ± 0.87**	**18.4 ± 0.89**		η_p^2 =0.177	η_p^2 = 0.004	η_p^2 =0.012
		**Sex**	**Treatment**		**Sex mean**	Treatment	Sex	Treatment*Sex
		male	2.07 ± 0.65	1.76 ± 0.61	**1.91 ± 0.45**	F(1,62)=1.61	F(1,62)=2.78	F(1,62)=0.61
	**UR**	female	3.58 ± 0.58	2.31 ± 0.63	**3.00 ± 0.43**	p=0.2092	p=0.1006	p=0.4382
		**Treatment mean**	**2.91 ± 0.44**	**2.03 ± 0.44**		η_p^2 =0.025	η_p^2 = 0.043	η_p^2 =0.010
**CT**	**Rc**	**Sex**	**Treatment**		**Sex mean**	Treatment	Sex	Treatment*Sex
		male	17.6 ± 1.45	17.0 ± 1.74	**17.3 ± 1.21**	F(1,25)=0.01	F(1,25)=0.65	F(1,25)=0.07
		female	18.6 ± 0.73	18.9 ± 2.30	**18.7 ± 1.24**	p=0.9229	p=0.4270	p=0.7952
		**Treatment mean**	**18.1 ± 1.21**	**17.9 ± 1.25**		η_p^2 =0.000	η_p^2 = 0.025	η_p^2 =0.003
**OLM**		**Location**	**Treatment**			Sex	Treatment	Location
	**FER** females	initial	0.78 ± 0.08	1.16 ± 0.24		F(1,28)=0.01	F(1,28)=3.52	F(1,28)=6.27
		changed	2.18 ± 0.55	1.22 ± 0.22		p=0.9333	p=0.0711	**p=0.0183**
						η_p^2 =0.000	η_p^2 = 0.112	η_p^2 =0.183
						Sex*Treatment	Sex*Location	Treatment*Loc
	**FER** males	initial	1.14 ± 0.14	1.10 ± 0.27		F(1,28)=0.48	F(1,28)=0.56	F(1,28)=7.99
		changed	2.13 ± 0.55	0.91 ± 0.14		p=0.4916	p=0.4605	**p=0.0086**
						η_p^2 =0.017	η_p^2 = 0.019	η_p^2 =0.222
						Sex*Treat*Loc, F(1,28)=0.03, p=0.8863		
**ART**		**Training**	**Treatment**			Sex	Treatment	Training
	**TC** females	before	7.14 ± 1.56	8.57 ± 2.74		F(1,27)=0.79	F(1,27)=0.002	F(1,27)=17.4
		after	3.63 ± 0.93	3.25 ± 0.60		p=0.3815	p=0.9643	**p=0.0003**
						η_p^2 =0.028	η_p^2 = 0.000	η_p^2 =0.392
						Sex*Treatment	Sex*Training	Treat*Train
	**TC** males	before	9.57 ± 1.46	7.36 ± 0.35		F(1,27)=0.23	F(1,27)=0.10	F(1,27)=0.15
		after	4.06 ± 0.77	5.19 ± 1.22		p=0.6362	p=0.7579	p=0.7046
						η_p^2 =0.008	η_p^2 = 0.004	η_p^2 =0.006
						Sex*Treat*Train, F(1,27)=1.74, p=0.1978		
		**Training**	**Treatment**			Sex	Treatment	Training
	**TR** females	before	16.2 ± 3.90	19.8 ± 2.60		F(1,27)=0.10	F(1,27)=0.22	F(1,27)=63.0
		after	3.57 ± 0.43	3.57 ± 0.53		p=0.7600	p=0.6447	**p<0.0001**
						η_p^2 =0.004	η_p^2 = 0.008	η_p^2 =0.70
						Sex*Treatment	Sex*Training	Treat*Train
	**TR** males	before	17.1 ± 3.17	14.3 ± 3.23		F(1,27)=0.27	F(1,27)=1.22	F(1,27)=0.10
		after	3.50 ± 0.55	6.13 ± 1.46		p=0.6096	p=0.2800	p=0.7508
						η_p^2 =0.010	η_p^2 = 0.043	η_p^2 =0.004
						Sex*Treat*Train, F(1,27)=1.94, p=0.1752		

Rof – rearing in open field, CT – cylinder test, OLM – object location memori test; ATR – adhesive tape removal test, SR – number of supported rearing, UR – number of unsupported rearing, Rc – number of rearings in cylinder, FER – frequency of entry ratio; TC – time-to-contac (s), TR – time-to-remove (s) the tape. Rof and CT are analyzed by two-way ANOVA; OLM and ATR are analyzed by repeated measure three-way ANOVA. η_p^2 - partial eta squared, 0.01 indicating a small, 0.06 a medium, and 0.14 a large effect size (https://effect-size-calculator.herokuapp.com/).

Supplementary Table 4. Values and statistics for neurochemical and molecular parameters measured by ELISA and qPCR, respectively.

	VALUES (M ± SEM)				STATISTICAL PARAMETERS		
Parameter		Treatment			Treatment	Sex	Interaction
	Sex	control	hypoxic	**Sex mean**			
**DA** (pg/mg)	male	72.9 ± 9.76	231 ± 30.2	**157 ± 20.4**	F(1,27)=25.8	F(1,27)=0.05	F(1,27)=0.23
	female	92.8 ± 18.2	223 ± 41.0	**158 ± 19.7**	**p<0.0001**	p=0.8271	p=0.6348
	**Treatment mean**	**83.4 ± 20.4**	**227 ± 19.7**		η_p^2 =0.487	η_p^2 = 0.002	η_p^2 =0.008
**NA** (pg/mg)	male	536 ± 21.4	571 ± 31.1	**555 ± 23.7**	F(1,27)=0.76	F(1,27)=0.56	F(1,27)=0.04
	female	518 ± 32.9	540 ± 40.6	**529 ± 22.9**	p=0.3015	p=0.4621	p=0.8499
	**Treatment mean**	**527 ± 23.7**	**555 ± 22.9**		η_p^2 =0.027	η_p^2 = 0.020	η_p^2 =0.001
**D1** gene hippocampus (AU)	male	210 ± 40,1	230 ± 28.8	**220 ± 31.7**	F(1,25)=0.01	F(1,25)=4.74	F(1,25)=0.35
	female	332 ± 52.2	301 ± 43.3	**316 ± 30.7**	p=0.9145	**p=0.0391**	p=0.5624
	**Treatment mean**	**271 ± 31.7**	**268 ± 30.7**		η_p^2 =0.000	η_p^2 = 0.159	η_p^2 =0.014
**D2** gene hippocampus (AU)	male	38.3 ± 9.39	34.5 ± 4.07	**36.5 ± 25.2**	F(1,23)=0.05	F(1,23)=9.51	F(1,23)=0.47
	female	139 ± 35.8	147 ± 44.1	**143 ± 22.6**	p=0.8199	**p=0.0052**	p=0.4986
	**Treatment mean**	**92.8 ± 24.2**	**98.6 ± 23.5**		η_p^2 =0.002	η_p^2 = 0.293	η_p^2 =0.020
**D1** gene thalamus (AU)	male	81.0 ± 8.57	111 ± 15.7	**95.9 ± 9.02**	F(1,25)=7.59	F(1,25)=0.01	F(1,25)=0.13
	female	77.8 ± 9.55	117 ± 9.17	**98.5 ± 8.73**	**p=0.0108**	p=0.9181	p=0.7262
	**Treatment mean**	**79.3 ± 9.02**	**114 ± 8.73**		η_p^2 =0.233	η_p^2 = 0.000	η_p^2 =0.005
**D2** gene thalamus (AU)	male	24.0 ± 14.4	80.8 ± 20.1	**56.5 ± 17.3**	F(1,23)=7.44	F(1,23)=6.95	F(1,23)=1.35
	female	79.9 ± 20.8	144 ± 26.9	**110 ± 17.8**	**p=0.0120**	**p=0.0148**	p=0.2573
	**Treatment mean**	**54.1 ± 17.9**	**108 ± 17.3**		η_p^2 =0.244	η_p^2 = 0.232	η_p^2 =0.055
**PKArs** gene thalamus (AU)	male	87.1 ± 4.41	109 ± 9.69	**98.8 ± 5.95**	F(1,27)=6.17	F(1,27)=0.04	F(1,27)=0.02
	female	90.0 ± 8.29	109 ± 8.71	**99.6 ± 5.75**	**p=0.0195**	p=0.8525	p=0.8760
	**Treatment mean**	**88.7 ± 5.95**	**109 ± 5.75**		η_p^2 =0.186	η_p^2 = 0.001	η_p^2 =0.001
**DARP-32** gene thalamus (AU)	male	84 ± 7.30	165 ± 36.4	**127 ± 18.3**	F(1,27)=5.56	F(1,27)=0.001	F(1,27)=0.67
	female	104 ± 15.2	143 ± 28.2	**124 ± 17.6**	**p=0.0258**	p=0.9728	p=0.4181
	**Treatment mean**	**94.7 ± 18.3**	**154 ± 17.6**		η_p^2 =0.171	η_p^2 = 0.000	η_p^2 =0.024
**IPP5** gene thalamus (AU)	male	102 ± 33.1	317 ± 61.3	**217 ± 31.0**	F(1,27)=20.0	F(1,27)=0.79	F(1,27)=0.26
	female	86.1 ± 12.9	257 ± 45.3	**171 ± 29.9**	**p=0.0001**	p=0.3823	p=0.6121
	**Treatment mean**	**93.7 ± 31.0**	**287 ± 29.9**		η_p^2 =0.426	η_p^2 = 0.028	η_p^2 =0.010

DA – dopamine, NA – noradrenaline, D1 - dopamine receptor 1, D2 - dopamine receptor 2, PKArs - regulatory subunit of protein kinase A, DARP-32 - dopamine- and cAMP-regulated phosphoprotein, IPP5 - inhibitor-5 of protein phosphatase 1. mRNA levels are expressed in arbitrary units (AU), i.e. normalized to a calibrator and relative to an average of the two endogenous references, as $2^{-\Delta\Delta Ct}$ x 100. η_p^2 - partial eta squared, 0.01 indicating a small, 0.06 a medium, and 0.14 a large effect size (https://effect-size-calculator.herokuapp.com/).
